# Preclinical Characterisation of PSMA/GRPR-Targeting Heterodimer [^68^Ga]Ga-BQ7812 for PET Diagnostic Imaging of Prostate Cancer: A Step towards Clinical Translation

**DOI:** 10.3390/cancers15020442

**Published:** 2023-01-10

**Authors:** Fanny Lundmark, Ayman Abouzayed, Sara S. Rinne, Vasiliy Timofeev, Nadezhda Sipkina, Maria Naan, Anastasia Kirichenko, Maria Vasyutina, Daria Ryzhkova, Vladimir Tolmachev, Ulrika Rosenström, Anna Orlova

**Affiliations:** 1 Department of Medicinal Chemistry, Uppsala University, 751 23 Uppsala, Sweden; 2 Personalized Medicine Centre, Almazov National Medical Research Centre, 2 Akkuratova Str., 197341 Saint Petersburg, Russia; 3 Preclinical and Translational Research Centre, Almazov National Medical Research Centre, 2 Akkuratova Str., 197341 Saint Petersburg, Russia; 4 Department of Nuclear Medicine and Radiation Technology with Clinic, Almazov National Medical Research Centre, 2 Akkuratova Str., 197341 Saint Petersburg, Russia; 5 Department of Immunology, Genetics and Pathology, Uppsala University, 751 23 Uppsala, Sweden; 6 Science for Life Laboratory, Department of Medicinal Chemistry, Uppsala University, 751 23 Uppsala, Sweden

**Keywords:** prostate cancer, PSMA, GRPR, heterodimer, PET imaging, diagnostic, molecular imaging, clinical translation

## Abstract

**Simple Summary:**

Prostate cancer continues to be the most frequently diagnosed form of cancer and the leading cause of cancer-related deaths among men. For a successful treatment plan and outcome, an early diagnosis, correct staging, and monitoring of treatment response are crucial. To improve this, a radiotracer could be used to target the two most abundant proteins overexpressed in prostate cancer: prostate-specific membrane antigen (PSMA) and gastrin-releasing peptide receptor (GRPR). To date, no such heterodimeric radiotracer is used in the clinic. In this study, we have preclinically characterized and evaluated a galium-68 labelled PSMA/GRPR-targeting radiotracer for PET imaging of prostate cancer. We hope that the findings from this study will be able to contribute to designing better heterodimeric ligands, promote clinical translation of a heterodimer, and serve as a step towards a first-in-human study.

**Abstract:**

The development of radioligands targeting prostate-specific membrane antigen (PSMA) and gastrin-releasing peptide receptor (GRPR) has shown promising results for the imaging and therapy of prostate cancer. However, studies have shown that tumors and metastases can express such targets heterogeneously. To overcome this issue and to improve protein binding, radioligands with the ability to bind both PSMA and GRPR have been developed. Herein, we present the preclinical characterization of [^68^Ga]Ga-BQ7812; a PSMA/GRPR-targeting radioligand for the diagnostic PET imaging of prostate cancer. This study aimed to evaluate [^68^Ga]Ga-BQ7812 to promote the translation of such imaging probes into the clinic. [^68^Ga]Ga-BQ7812 demonstrated rapid and specific binding to both targets in a PSMA/GRPR-expressing PC3-pip cell line. Results from the biodistribution study in PC3-pip xenografted mice showed specific binding to both targets, with the highest activity uptake at 1 h pi in tumor (PSMA+/GRPR+, 10.4 ± 1.0% IA/g), kidneys (PSMA+, 45 ± 16% IA/g), and pancreas (GRPR+, 5.6 ± 0.7% IA/g). At 3h pi, increased tumour-to-organ ratios could be seen due to higher retention in the tumor compared with other PSMA or GRPR-expressing organs. These results, together with low toxicity and an acceptable estimated dosimetry profile (total effective dose = 0.0083 mSv/MBq), support the clinical translation of [^68^Ga]Ga-BQ7812 and represent a step towards its first clinical trial.

## 1. Introduction

Over the past decade, a radiopharmacutical research has focused on the development of radioligands against PCa-specific biomarkers. These efforts have recently resulted in an improved diagnosis and treatment of PCa. Two of the most investigated molecular targets in PCa are the prostate-specific membrane antigen (PSMA, also known as glutamate carboxypeptidase II) and the gastrin-releasing peptide receptor (GRPR). Both are overexpressed in malignant PCa cells compared with benign prostate tissue and healthy organs, making them suitable targets for diagnostic and therapeutic radiopharmaceuticals [1,2].

Indeed, the expression of PSMA in malignant PCa cells is significantly higher (100–1000 fold) compared to the endogenous expression found in proximal renal tubules, small intestine, salivary glands, and benign prostate tissue. Such over-expression is observed throughout all stages of PCa but tends to increase with the disease progression [3,4]. Small urea-based pseudo-peptide inhibitors are the most commonly studied type of PSMA-targeting radioligands for both diagnostic and therapeutic applications. Several studies have focused on optimising the molecular design of such tracers, resulting in novel compounds with improved targeting properties [5,6,7]. Consequently, numerous clinical studies of PSMA-targeting radioligands for imaging and radioligand therapy with promising results are also ongoing [8]: [^68^Ga]Ga-PSMA-11 and [^18^F]F-DCFPyL for positron emission tomography (PET) imaging of PSMA-positive lesions, and the theranostic pair [^68^Ga]Ga-PSMA-617/[^177^Lu]Lu-PSMA-617 for treatment of progressive PSMA-positive metastatic castration-resistant PCa are three PSMA-binding radioligands approved by the Food and Drug Administration (FDA) [9,10,11,12].

GRPR is a G-protein coupled receptor belonging to the bombesin receptor family and is normally expressed in the pancreas. Only low levels of GRPR have been detected in the GI tract and, most importantly, in benign prostate tissue [13]. Stimulation of GRPR promotes smooth muscle contraction in the GI tract and has been shown to increase cancer cell proliferation and tumour growth [14,15]. Importantly, the receptor is not only significantly over-expressed in the majority of PCa cases (62–100%), but also in other cancer types, such as breast, lung, head and neck, pancreatic cancers, and brain malignant tumours [16,17]. Over-expression of GRPR in PCa has been found in both primary tumours as well as lymph nodes and bone metastasis. In contrast to PSMA, GRPR levels might decrease with PCa progression due to its androgen-dependent expression, [3,13,18]. Several agonistic and antagonistic GRPR-targeting radioligands have been developed and evaluated throughout the years. The first-generation GRPR-targeting agonists showed some promise but the second-generation antagonists were found to be more beneficial; they not only demonstrated less off-target effects but also a higher tumour uptake, more advantageous pharmacokinetics, and higher tumour-to-background ratios [19,20]. Antagonists for the imaging of GRPR-expressing PCa, such as RM2 for PET ([^68^Ga]Ga-RM2) and single-photon emission computed tomography (SPECT) ([^111^In]In-RM2), have shown promising preclinical results. [^68^Ga]Ga-RM2 has been further evaluated in the clinic, demonstrating its ability to safely image both primary tumours and metastatic lesions [21,22].

The development of radioligands with the ability to target either PSMA or GRPR has significantly contributed to the theranostic field of PCa. However, their use is dependent on the homogeneous expression of one target alone. Therefore, any variation, such as lack of membrane localisation or heterogenous expression of the targets, would negatively affect their potential. Heterogenous expression of the two targets was confirmed in a direct comparison of [^68^Ga]Ga-RM2 binding to GRPR and [^68^Ga]Ga-PSMA-11 binding to PSMA in patients with biochemically recurrent PCa [23]. It has also been demonstrated that both primary tumours and distant metastases, independent of Gleason score, histological subtype, or localization of metastases, could lack large areas of PSMA expression [24]. Based on this, a heterodimeric radiotracer with the ability to target both GRPR and PSMA could be beneficial.

**Figure 1:**
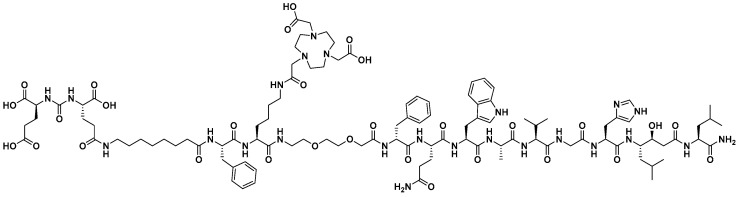
Chemical structure of the PSMA/GRPR-targeting heterodimeric radiotracer BQ7812.

Since the first PSMA/GRPR-heterodimers were published in 2014 [25,26], several more have been developed with the aim to increase the imaging sensitivity and specificity compared to their respective monomers [27,28,29,30,31,32,33,34]. Up to now, the only PSMA/GRPR-heterodimer that has been clinically evaluated is [^68^Ga]Ga-DOTA-PSMA(Inhibitor)-Lys_3_-Bombesin. While its safe toxicology and dosimetry profiles have been shown in healthy volunteers, more clinical data are still needed [35]. In 2020, our group reported on the pre-clinical evaluation of an [^111^In]In-BQ7812 heterodimer for PSMA/GRPR-targeting (Figure 1), which showed promising characteristics in SPECT imaging of PCa in murine model [32]. Another imaging modality that could be used for cancer diagnostics is PET, which has many advantages compared to SPECT: it features a higher sensitivity and resolution for easier detection of small lesions (below 1 cm) as well as a superior quantitative capability for improved therapy planning. Gallium-68 is a common radionuclide used for PET imaging. It is generator-produced, making it easy to access and relatively cheap. The gallium-68 half-life of 68 min is also very suitable for the use of peptide-based targeting agents due to their kinetics [36,37,38]. To contribute to the development and clinical translation of PSMA/GRPR-targeting heterodimers for PET imaging of PCa, herein, we present the preclinical evaluation of [^68^Ga]Ga-BQ7812.

## 2. Materials and Methods

### 2.1. Synthesis

Synthesis of BQ7812 was based on a previously described procedure [32]. Briefly, the compound was synthesised by solid-phase peptide synthesis (SPPS) on Rink amide resin (loading 0.69 mmol/g) using Oxyma Pure and DIC as coupling reagents and DIPEA as a base. All couplings were performed in DMF for at least 3 h and the final compound was purified using preparative reverse-phase high-performance liquid chromatography (RP-HPLC). The purity and identity of the product were confirmed using analytical HPLC-MS (details and chromatograms in Figures S1 and S2 in the Supplementary Material). Calculated [M+2H]^2+^ and [M+3H]^3+^: 1132.1 and 755.1. Observed [M+2H]^2+^ and [M+3H]^3+^: 1132.0 and 754.9. The final compound was freeze-dried, dissolved in Chelex-treated Milli-Q water, and stored at −20 °C. 

### 2.2. Gallium-68 Labelling and Stability

Gallium-68 was eluted from a ^68^Ge/Ga-generator (Cyclotron Co., Obninsk, Russia) using 0.1 M metal-free hydrochloric acid. To a solution of BQ7812 (2 nmol) in sodium acetate buffer (200 μL, 1.25 M, pH 3.6), gallium-68 (20 MBq) was added and the reaction was left for 10 min at 85 °C. Instant thin-layer chromatography (ITLC) was used to analyse the radiochemical yield using 0.2 M citric acid as an eluent and [^68^Ga]Ga-BQ7812 was used without further purification. 

To evaluate the stability, [^68^Ga]Ga-BQ7812 was incubated in human serum at 37 °C or PBS at room temperature. [^68^Ga]Ga-BQ7812 was also challenged with 1000-fold excess of EDTA. After 1 h, samples were taken from all solutions and analysed by ITLC. 

For clinical translation, BQ7812 (2 nmol) was labelled with 1.5 GBq of gallium-68 eluate in sodium acetate buffer (0.65 mL, 2 M, pH 4.5) for 12 min at 90 °C. ITLC was used to analyse the radiochemical yield (eluent: NaOAc/MeOH (1:1)) and the presence of colloids (eluent: 0.2 M sodium citrate). For toxicology studies, BQ7812 (2 nmol) was labelled with 100 MBq of gallium-68 using the same procedure.

### 2.3. In Vitro Characterisation

In vitro experiments were carried out using the PSMA- and GRPR-expressing human prostate carcinoma PC3-pip cell line (obtained from Prof. Martin Pomper, The Johns Hopkins University, Baltimore, MD, USA). Cells (5–7 × 10^5^ cells/vial) were seeded 24–48 h before the experiments. All experiments were performed in triplicate. 

#### 2.3.1. Specific Binding

To evaluate the in vitro specific binding of [^68^Ga]Ga-BQ7812 to PSMA and GRPR, approximately 3 × 10^6^/well PC3-pip cells were seeded in a 12-well plate. To block the targets, cells were incubated at room temperature for 15 min with an excess (750 nM/well) of PSMA-11 (to block PSMA) and/or NOTA-PEG_2_-RM26 (to block GRPR). Thereafter, [^68^Ga]Ga-BQ7812 (20 nM/well) was added to all wells and cells were incubated at 37 °C. After 1 h, cells were collected, and the radioactive content was measured using an automated gamma counter. 

#### 2.3.2. Cellular Processing

The cellular processing was studied by incubating PC3-pip cells seeded in 3 cm Petri dishes (3 × 10^6^/dish) with [^68^Ga]Ga-BQ7812 (1 nM/well) at 37 °C. At selected time points (1 and 3 h), cells were collected, and the total cell-associated activity was measured using an automated gamma counter. At 3 h, the membrane-bound and internalised fractions were collected separately using the acid and base method described earlier [32]. 

### 2.4. Ex Vivo and In Vivo Experiments

Ex vivo experiments and in vivo imaging were carried out using BALB/c nu/nu mice bearing PC3-pip xenografts. All studies were performed according to the guidelines of the Declaration of Helsinki and approved by the Ethics Committee for Animal Research in Uppsala, Sweden (5.8.18-00473/2021. Approved on 26 February 2021).

#### 2.4.1. Biodistribution

To study the biodistribution profile over time, each mouse was intravenously injected with 40 pmol of [^68^Ga]Ga-BQ7812 (300 kBq/mouse in the 1 h pi group and 700 kBq/mouse in the 3 h pi group) dissolved in a total volume of 100 μL 1% Bovine Serum Albumin (BSA) in PBS. There were 4 mice in each group. At 1 and 3 h pi, mice were sacrificed after an intraperitoneal injection of a Ketalar–Rompun solution (20 μL/g body weight) consisting of Ketalar (10 mg/mL) and Rompun (1 mg/mL), followed by heart puncture. To demonstrate specific binding in vivo, mice were co-injected with non-labelled NOTA-PEG_2_-RM26 (5 nmol) or non-labelled PSMA-11 (5 nmol) to block GRPR and PSMA respectively. After 1 h, mice were sacrificed according to the procedure described above and the uptake of [^68^Ga]Ga-BQ7812 was measured in organs of interest. The average animal weight was 17.4 ± 0.96 g.

#### 2.4.2. Imaging

Whole body nanoPETimaging of PC3-pip xenografted mice using a nanoScan PET/MR1 3T camera (Mediso Medical Imaging System Ltd., Budapest, Hungary) was conducted by the injection of [^68^Ga]Ga-BQ7812 (100 pmol, 8 MBq) dissolved in a total volume of 100 μL 1% BSA. Immediately after the PET scan, a CT scan was performed using a nanoScan SPECT/CT camera (Mediso Medical Imaging System Ltd., Budapest, Hungary) with the same bed. In blocked group, mice were co-injected with non-labelled NOTA-PEG_2_-RM26 (5 nmol) and non-labelled PSMA-11 (5 nmol), in addition to the gallium-68 labelled heterodimer. Imaging of the non-blocked (*n* = 2) and the PSMA/GRPR-blocked (*n* = 2) groups was performed at 1 and 3 h pi. Reconstruction of the PET/CT scans was conducted using Nucline nanoScan 3.04.014.0000 software.

#### 2.4.3. Dosimetry

For dosimetry evaluation, NMRI mice were injected with [^68^Ga]Ga-BQ7812 (40 pmol, 150–720 kBq) dissolved in a total volume of 100 μL 1% BSA. At 0.5, 1, 2, and 4 h pi, mice were sacrificed according to the procedure described above and the biodistribution was evaluated based on the uptake of [^68^Ga]Ga-BQ7812 in the organs of interest. The average animal weight was 27.4 ± 1.6 g and OLINDA/EXM 1.0 software (adult male phantom) was used to estimate the absorbed doses.

The well-established “percent kg/g method” using Equation (1) was used to upscale the uptake values in mice to be able to evaluate the dosimetry in humans. The organ weights of the reference adult male were used to calculate the organ uptake in humans and the area under the curve of exponential fits for human time–activity curves was used to calculate the residence times.

(%IA/organ)_human_ = [(%IA/g)_animal_ × (kBqTBweight)_animal_ × (g_organ_/(kBqTBweight)_human_] (1)

#### 2.4.4. Toxicology

Acute (within 24 h pi) and subacute (up to 28 days pi) toxicity for BQ7812 (Pepmic, China) and [^68^Ga]Ga-BQ7812 (labelled in-house) were studied at NAMRC, Russia, in Wistar rats (Protocol PZ_22_6_Kirichenko AS_V1, approved by ethical committee on animal research of NAMRC on 7 June 2022). The animals were kept in a barrier-type animal facility under standard housing conditions [39]. Rats (*n* = 15 per each group, average weight 427 g) were iv injected (single bolus) with 10.3 μg of BQ7812 in 0.2 mL of saline, or with [^68^Ga]Ga-BQ7812 (10 MBq, 10.3 μg), or with 0.2 mL saline, or with 0.2 mL of sodium acetate buffer. The dose of heterodimer for toxicological studies was calculated based on cross-species dose conversion using body surface area scaling: the maximum dose for the clinical study was chosen to be 300 µg of heterodimer per 75 kg patient, or 4 mg/kg, which corresponds to [(4 µg × 427 g)/1000 g] × 6 = 10.3 µg per rat [40]. Ten rats from each group were euthanized at 24 h pi and five rats at 28 days pi. The body weight of the rats was controlled at baseline, at 24 h after administration for the acute toxicity group, and every second day for the subacute toxicity group. Biochemical blood tests on alanine aminotransferase, albumin, aspartate aminotransferase, bilirubin, glucose, creatinine, urea, the total amount of proteins, cholesterol, and ALT were performed before the euthanasia. Necropsy of the experimental rats was performed, and the weights of the brain, heart, lungs, liver, adrenal glands, kidneys, spleen, testes, and thymus were measured.

#### 2.4.5. Statistical Analysis

Obtained values are presented as average with standard deviation. Data were assessed either by an unpaired, two-tailed *t*-test or by one-way ANOVA with Bonferroni correction for multiple comparisons using GraphPad Prism (version 6 for Windows GraphPad Software) in order to determine significant differences (*p* < 0.05).

## 3. Results

### 3.1. Synthesis and Radiolabelling

BQ7812 (Figure 1) was successfully synthesised and radiolabelled with gallium-68 in high radiochemical yields (97–99%). Only a minor release of free gallium-68 was detected by ITLC after incubation of [^68^Ga]Ga-BQ7812 in PBS or a 1000-fold molar excess EDTA for 1 h. [^68^Ga]Ga-BQ7812 was stable in human serum when incubated at 37 °C for 1 h and no release of free gallium-68 was detected after analysis by ITLC.

### 3.2. In Vitro Characterisation

**Figure 2:**
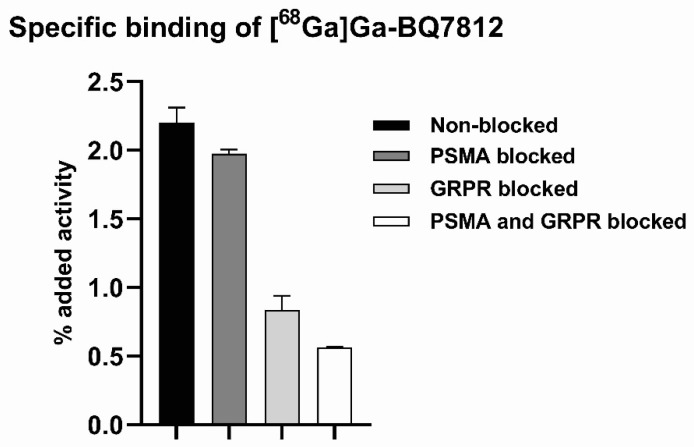
In vitro binding specificity of [^68^Ga]Ga-BQ7812. Blocking was performed by the addition of non-labelled PSMA-11 and/or non-labelled NOTA-PEG_2_-RM26 (500 nM/well). Statistical analysis was done using one-way ANOVA with Bonferroni’s test corrected for multiple comparisons. A statistically significant difference was seen between all groups.

Results from the in vitro binding specificity study demonstrated specific binding of [^68^Ga]Ga-BQ7812 towards PSMA and GRPR (Figure 2). Pre-saturation of PSMA, GRPR, or PSMA and GRPR resulted in a significant decrease (*p* < 0.05) in activity uptake compared to naive cells. The uptake of [^68^Ga]Ga-BQ7812 in cells pre-saturated with both PSMA- and GRPR-blocking agents was significantly lower compared to the uptake of activity in cells pre-saturated with either a PSMA- or GRPR-blocking agent. This demonstrated that both targets contribute to the uptake of the heterodimeric radioligand. 

**Figure 3:**
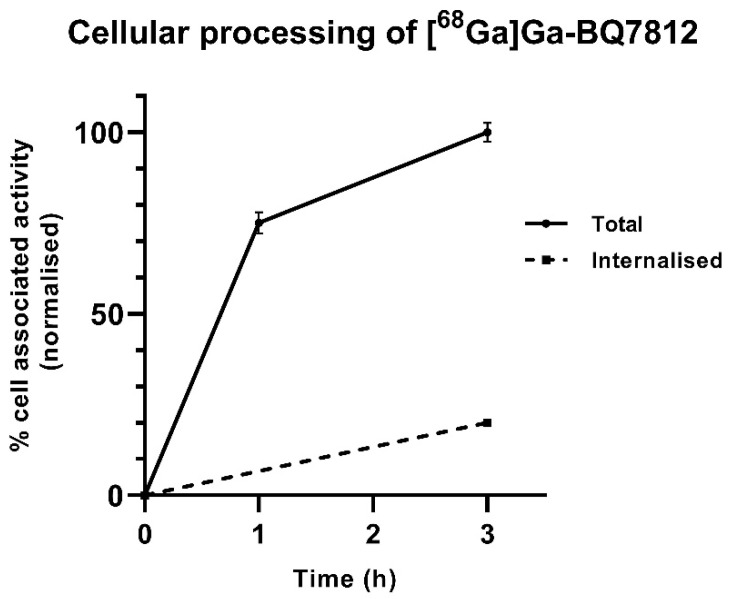
Cellular processing of [^68^Ga]Ga-BQ7812. Values are normalized to the highest uptake in the experiment.

Results from the cellular processing of [^68^Ga]Ga-BQ7812 showed rapid binding to the targets (Figure 3). Of the total cell-associated activity at 3 h, approximately 20% was internalised and 80% membrane-bound, which is in accordance with the cellular processing data reported for [^111^In]In-BQ7812 [32].

### 3.3. In Vivo Characterisation

**Figure 4:**
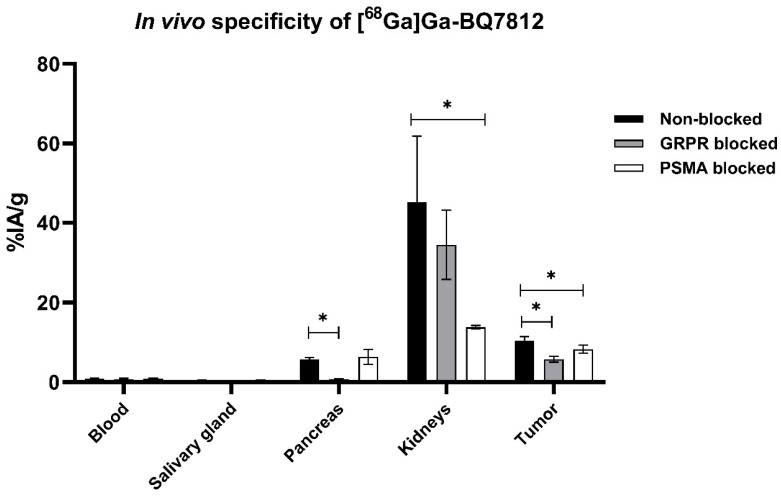
In vivo binding specificity of [^68^Ga]Ga-BQ7812 towards PSMA and GRPR at 1 h pi in PC3-pip tumour-bearing mice. Co-injection of non-labelled NOTA-PEG_2_-RM26 (5 nmol/animal) or non-labelled PSMA-11 (5 nmol/animal) to block GRPR and PSMA respectively. Statistical analysis was done using one-way ANOVA with Bonferroni’s test corrected for multiple comparisons. * Statistically significant (*p* < 0.05) difference in organ uptake.

Specific binding to PSMA and GRPR was tested in vivo in mice bearing the PC3-pip xenografts. Mice in the PSMA-blocked group demonstrated a statistically significantly lower uptake of [^68^Ga]Ga-BQ7812 in the tumour and kidneys compared with the non-blocked group. The uptake of [^68^Ga]Ga-BQ7812 in the GRPR-blocked group was statistically significantly lower in the tumour and the pancreas compared with the non-blocked group. These results demonstrate the specific binding of [^68^Ga]Ga-BQ7812 to both PSMA and GRPR since the activity uptake in the tumours as well as in PSMA- (kidneys) and GRPR-expressing (pancreas) normal organs is significantly decreased when blocking the targets (Figure 4).

**Figure 5:**
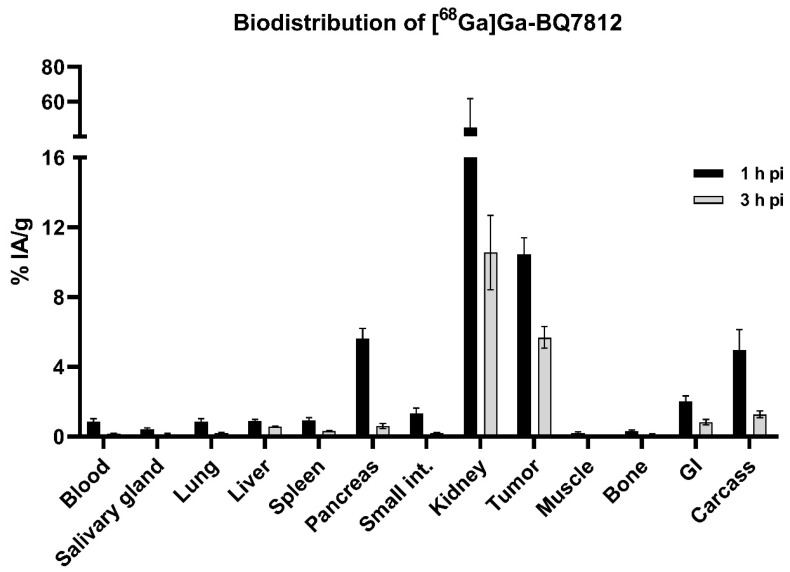
In vivo biodistribution of [^68^Ga]Ga-BQ7812 in PC3-pip tumour-bearing mice at 1 and 3 h post-injection showing the uptake activity in specified organs.

**Figure 6:**
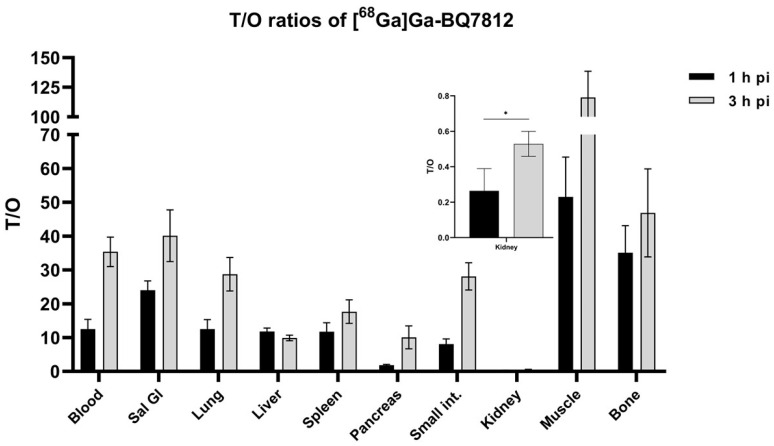
In vivo biodistribution of [^68^Ga]Ga-BQ7812 in PC3-pip tumour-bearing mice at 1 and 3 h post-injection showing the Tumour-to-organ ratios (T/O). Statistical analysis was done using unpaired, two-tailed *t*-test. * Statistically significant (*p* < 0.05) difference in tumor to kidney ratio.

The biodistribution profile of [^68^Ga]Ga-BQ7812 was studied at 1 and 3 h pi in BALB/c nu/nu mice bearing PC3-pip xenografts. The activity uptake in the different organs is presented in Figure 5 (details in Table S1 in Supplementary Materials). The biodistribution pattern was characterised by an elevated uptake in the tumour (10.4 ± 1.0% IA/g), kidneys (45 ± 16% IA/g), and pancreas (5.6 ± 0.6% IA/g) at 1 h pi. The uptake of activity in remaining organs at 1 h pi was approximately 1% IA/g or below. At 3 h pi, the activity uptake had decreased in all organs and an elevated uptake of activity could only be seen in the tumour (5.7 ± 0.6% IA/g) and in the kidneys (12 ± 6% IA/g). The decrease of activity uptake between 1 and 3 h pi was higher in the kidneys and pancreas compared with the tumour. At 3 h pi, the activity uptake in the tumour was around 55% of the activity uptake at 1 h pi, whereas the activity uptake in the kidneys and pancreas was around 26% and 11% compared to the activity uptake in the respective organ at 1 h pi. Tumour-to-organ ratios (T/O) are presented in Figure 6 (details in Table S1 in Supplementary Materials). T/O increased from 1 to 3 h pi for all organs except the liver and the highest ratios were T/muscle (116 ± 22) and bone (47 ± 13).

**Figure 7:**
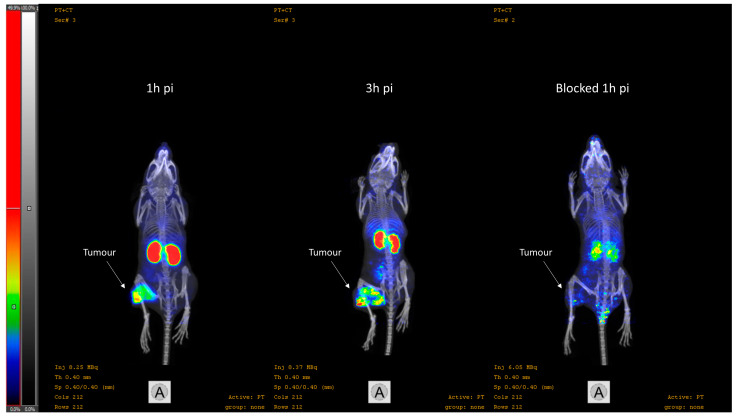
Representative nanoScan PET/CT images of PC3-pip xenografted mice injected with [^68^Ga]Ga-BQ7812 (100 pmol, 8 MBq). To the left: 1 h pi.; in the middle: 3 h pi.; to the right: PSMA and GRPR blocked at 1 h pi.

The nanoScan PET/CT images (Figure 7) confirmed the results from the ex vivo measurements of the biodistribution study. An elevated activity uptake could be visualised in the kidneys and the tumour at 1 and 3 h pi. Upon blocking of PSMA and GRPR, the tumour is no longer visualised and the kidneys are not as well defined. 

To evaluate the dosimetry, the absorbed dose for men in each organ (Table 1) was estimated based on the ex vivo data from mice (Table S1) using Equation (1). Organs with the highest estimated absorbed doses were the kidneys (0.0660 mGy/MBq), the heart wall (0.0265 mGy/MBq), and the lower large intestine wall (0.0250 mGy/MBq). The absorbed doses were below 0.02 mSv/MBq in the remaining organs and tissues. The total effective dose was found to be 0.0083 mSv/MBq.

The results of the preclinical toxicological studies showed no changes in clinical or biochemical parameters in experimental and control groups. Pathomorphological examination of adrenal glands, liver, brain, lungs, thymus, spleen, heart, testes, kidneys, and bladder was performed, and mild morphological changes were revealed in the studied organs of the experimental and control groups both for BQ7812 and [^68^Ga]Ga-BQ7812. The absence of significant differences in morphological changes between experimental and control groups was considered as an absence of toxicity of the studied radiopharmaceutical and the non-labelled peptide.

## 4. Discussion

As PCa continues to be the most frequently diagnosed cancer and one of the main causes for cancer-related deaths amongst men, more focus on the development of new pharmaceutical treatments and diagnostic procedures for PCa is warranted [40,41,42]. Radionuclide molecular imaging using PET in combination with CT or MRI is a multimodal technology with high precision and sensitivity which allows for the detection of microscale lesions. The use of radioligands with the ability to bind to PCa-specific biomarkers makes molecular imaging a promising approach for both diagnosis and staging as well as monitoring treatment outcomes. The two most commonly expressed and investigated PCa-specific biomarkers are PSMA and GRPR. Radioligands binding to either of these targets have shown promising results [9,10,11,12,21,22]. However, differences in the expression of PSMA and GRPR have been found both within the same patient and between different patients. The heterogeneous expression increases the risk of false-negative diagnoses and incorrect staging which could decrease the chance of successful treatment [23,24,43]. To overcome this, attempts have been made to develop bispecific radioligands with the ability to target both PSMA and GRPR. Such PSMA/GRPR-radioligands could increase the chance of specific binding to tumours and thereby promote the detection accuracy for patients with a non-homogenous expression of either of the targets. It could also be relevant for therapy to improve tracer delivery and thereby promote dose distribution and malignant cells killing. Promising results for this rather new approach of using PSMA/GRPR-heterodimeric radiotracers have been presented in the literature. However, their translation into the clinic is yet to occur. 

Herein, we present the results from the preclinical evaluation of the heterodimeric PSMA/GRPR-radioligand [^68^Ga]Ga-BQ7812 for PET/CT-imaging of PCa. Results show that [^68^Ga]Ga-BQ7812 binds specifically to PSMA and GRPR both in vitro and in vivo since pre-saturation of the targets leads to a significant decrease in activity uptake (Figure 2, Figure 4, and Figure 7). The biodistribution pattern showed specific activity uptake in the tumour, as well as elevated uptake in the kidneys and pancreas at 1 h pi. This was expected due to the endogenous PSMA and GRPR expression in these organs. The activity uptake decreased with time in all organs and was at 3 h pi highest in the kidneys (11.7 ± 5.8% IA/g) followed by the tumour (5.7 ± 0.62% IA/g). However, the decrease of activity in the tumour was lower compared with the activity decrease in healthy organs. At 3 h pi, the uptake of [^68^Ga]Ga-BQ7812 in the tumour was 55% of the uptake at 1 h pi while it was only 26% and 11% in the kidneys and pancreas respectively. This could also be seen by the significantly increased tumour to kidney and tumour to pancreas ratios between 1 and 3 h pi (Figure 6). These results indicate better retention in the tumour compared to the PSMA-expressing kidneys and the GRPR-expressing pancreas which could be a result of increased avidity due to the dual targeting. Despite better activity retention in tumors than in kidneys, the biodistribution profile of the studied heterodimer is not favorable for targeting radiotherapy if be labelled with therapeutic radionuclides, e.g., luthetium-177. Tumor to kidney ratios for BQ7812 were below 0.5 at all studied time points when labelled either with gallium-68 or indium-111 [32]. However, the first-generation heterodimer reported by our group, BQ7800, which had 10-fold worse affinity to PSMA, demonstrated equal activity uptake in tumors and in kidneys in vivo [31,32]. This observation might be useful for molecular design of heterodimer for targeted radiotherapy. Overall, the biodistribution pattern of [^68^Ga]Ga-BQ7812 was similar to [^111^In]In-BQ7812 except for a lower activity uptake and retention in the liver when the ligand was labelled with gallium-68 compared with indium-111 [32]. 

Even though the PSMA/GRPR-heterodimeric compound BQ7812 has been labelled with indium-111 and preclinically evaluated in a previous study, it is important to re-evaluate the ligand when another radiolabel is used. Different labelling conditions for gallium-68 and indium-111 may affect the quality and reproducibility. Results from the labelling and stability experiments did, however, indicate stable [^68^Ga][Ga-NOTA]-complex and no radiolysis of the labelled ligand. The good match of the gallium-68 half-life with the fast kinetics of peptide-based imaging probes is one reason why gallium-68 could be a more suitable radiolabel for this type of PSMA/GRPR-targeting heterodimeric peptide-based radioligands than indium-111 (t½ gallium-68: 68 min, t½ indium-111: 2.8 days). An additional advantage of the gallium-68 label is that it is generator-produced and thereby relatively cheap and easily accessible at the majority of PET centres around the world. Finally, gallium-68 enables PET-imaging in the hospital, which can provide higher resolution and sensitivity compared with SPECT and hence facilitate the detection of smaller PCa lesions [36,37,38].

As the aim of this study was to bridge the gap between preclinical and clinical use of PSMA/GRPR-heterodimeric radiotracers, toxicology and dosimetry were evaluated. The estimated effective dose from the dosimetry study was within the same range as the doses of the majority of other gallium-68 labelled tracers reported in the literature (Table 2). It is, however, worth noting that some of the reported effective doses in Table 2 are based on clinical patient data and some are estimated values from animal studies. A direct comparison of the values is therefore not entirely possible. The estimated effective dose for [^68^Ga]Ga-BQ7812 (0.0083 mSv/MBq) in this study would result in a total dose per scan of 1.66–2.49 mSv after injection of 200–300 MBq, which is considered an acceptable value. Results from the toxicological evaluation showed no morphological changes in either group (experimental or control) indicating a safe toxicological profile of BQ7812 and [^68^Ga]Ga-BQ7812 in rats. Taken together, from a toxicity, biodistribution, and radiation dosimetry point of view, [^68^Ga]Ga-BQ7812 could be considered a promising candidate for PET-imaging of PSMA/GRPR-expression in the clinics.

## 5. Conclusions

To conclude, we have presented the preclinical evaluation of the PSMA/GRPR-targeting heterodimeric radioligand, [^68^Ga]Ga-BQ7812, for the PET-imaging of PCa. BQ7812 could be labelled with gallium-68 in high radio-chemical yields with good quality and reproducibility. [^68^Ga]Ga-BQ7812 demonstrated specific and rapid binding to its targets and its biodistribution pattern was characterised by an initial elevated activity uptake in the tumour (PSMA/GRPR positive), kidneys (PSMA positive), and pancreas (GRPR positive). Uptake in the kidney and pancreas cleared significantly faster than the uptake in the tumour. [^68^Ga]Ga-BQ7812 demonstrated an acceptable and comparable estimated effective dose to other gallium-68 labelled tracers and a safe toxicology profile. Overall, this study supports clinical translation and marks a first exploratory clinical trial for [^68^Ga]Ga-BQ7812. Moreover, the results could contribute to the further development of PSMA/GRPR-targeting heterodimeric radioligands.

## 6. Patents

A patent application No. 2002110661 was submitted 19 April 2022.

## Figures and Tables

**Table 1:** The absorbed dose (mGy/MBq) of [^68^Ga]Ga-BQ7812 in each of the targeted organs.

Target Organ	Absorbed Dose (mGy/MBq)
Adrenals	0.0043
Brain	0.0007
Breasts	0.0030
Gallbladder wall	0.0042
Lower large intestine wall	0.0250
Small intestine wall	0.0199
Stomach wall	0.0048
Upper large intestines LI wall	0.0111
Heart wall	0.0265
Kidneys	0.0660
Liver	0.0044
Lungs	0.0041
Muscle	0.0024
Ovaries	0.0045
Pancreas	0.0186
Red marrow	0.0057
Osteogenic cells	0.0080
Skin	0.0026
Spleen	0.0033
Testes	0.0030
Thymus	0.0038
Thyroid	0.0030
Urinary bladder wall	0.0036
Uterus	0.0042
Total body	0.0044
Effective dose equivalent (mSv/MBq)	0.0124
Effective dose (mSv/MBq)	0.0083

**Table 2:** Effective dose (mSv/MBq) of ^68^Ga-labelled radiotracers reported in the literature.

Tracer	Effective Dose (mSv/MBq)	Reference
[^68^Ga]Ga-BQ7812	0.0083	This study
[^68^Ga]Ga-DOTA-MGS5	0.01	[44]
[^68^Ga]Ga-iPSMA-Lys^3^	0.02	[35]
[^68^Ga]Ga-NODAGA-LM3	0.026	[45]
[^68^Ga]Ga-DOTA SA FAPi	0.011	[46]
[^68^Ga]Ga-FAPI-74	0.016	[47]
[^68^Ga]Ga-SB3	0.014	[48]
[^68^Ga]Ga-RM26	0.066	[49]

## Data Availability

The data generated during the current study are available from the corresponding author upon reasonable request.

## Supplementary Materials

The following supporting information can be downloaded at: https://www.mdpi.com/article/10.3390/cancers15020442/s1, General information for chemical syntheses: Instruments and Equipment, Chemicals and Solvents; Figure S1. Analytical HPLC chromatogram of BQ7812. UV-detection at 214 and 254 nm; Figure S2. MS-chromatogram of BQ7812 with observed [M+2H]^2+^ = 1132.0 and [M+3H]^3+^ = 754.9; Table S1. Ex vivo biodistribution of [68Ga]Ga-BQ7812 (40 pmol/animal, 30 kBq) in BALB/c nu/nu PC3-pip xenografted tumour bearing mice at 1 and 3 h pi. Data are presented as average ± standard deviation. To the left; Uptake of activity was calculated as percent injected dose per tissue weight (% ID/g), To the right; tumour-to-organ ratios.

## References

[1] Ceci F., Castellucci P., Cerci J.J., Fanti S. (2017). New aspects of molecular imaging in prostate cancer. Methods.

[2] Manafi-Farid R., Ranjbar S., Jamshidi Araghi Z., Pilz J., Schweighofer-Zwink G., Pirich C., Beheshti M. (2021). Molecular Imaging in Primary Staging of Prostate Cancer Patients: Current Aspects and Future Trends. Cancers.

[3] Barve A., Jin W., Cheng K. (2014). Prostate cancer relevant antigens and enzymes for targeted drug delivery. J. Control. Release.

[4] Donin N.M., Reiter R.E. (2017). Why Targeting PSMA Is a Game Changer in the Management of Prostate Cancer. J. Nucl. Med..

[5] Kopka K., Benešová M., Bařinka C., Haberkorn U., Babich J. (2017). Glu-ureido-based inhibitors of prostate-specific membrane antigen: Lessons learned during the development of a novel class of low-molecular-weight theranostic radiotracers. J. Nucl. Med..

[6] Shahrokhi P., Masteri Farahani A., Tamaddondar M., Rezazadeh F. (2021). The utility of radiolabeled PSMA ligands for tumor imaging. Chem. Biol. Drug Des..

[7] Lundmark F., Olanders G., Rinne S.S., Abouzayed A., Orlova A., Rosenström U. (2022). Design, Synthesis, and Evaluation of Linker-Optimised PSMA-Targeting Radioligands. Pharmaceutics.

[8] Jones W., Griffiths K., Barata P.C., Paller C.J. (2020). PSMA theranostics: Review of the current status of PSMA-targeted imaging and radioligand therapy. Cancers.

[9] FDA FDA Approves First PSMA-Targeted PET Imaging Drug for Men with Prostate Cancer. https://www.fda.gov/news-events/press-announcements/fda-approves-first-psma-targeted-pet-imaging-drug-men-prostate-cancer.

[10] Novartis Novartis PluvictoTM Approved by FDA as First Targeted Radioligand Therapy for Treatment of Progressive, PSMA Positive Metastatic Castration-Resistant Prostate Cancer. https://www.novartis.com/news/media-releases/novartis-pluvictotm-approved-fda-first-targeted-radioligand-therapy-treatment-progressive-psma-positive-metastatic-castration-resistant-prostate-cancer.

[11] (2021). Newsline: FDA Approves 18F-DCFPyL PET Agent in Prostate Cancer. J. Nucl. Med..

[12] Sun J., Lin Y., Wei X., Ouyang J., Huang Y., Ling Z. (2021). Performance of 18F-DCFPyL PET/CT Imaging in Early Detection of Biochemically Recurrent Prostate Cancer: A Systematic Review and Meta-Analysis. Front. Oncol..

[13] Markwalder R., Reubi J.C. (1999). Gastrin-releasing peptide receptors in the human prostate: Relation to neoplastic transformation. Cancer Res..

[14] Elshafae S.M., Hassan B.B., Supsavhad W., Dirksen W.P., Camiener R.Y., Ding H., Tweedle M.F., Rosol T.J. (2016). Gastrin-releasing peptide receptor (GRPr) promotes EMT, growth, and invasion in canine prostate cancer. Prostate.

[15] Reubi J.C. (2003). Peptide Receptors as Molecular Targets for Cancer Diagnosis and Therapy. Endocr. Rev..

[16] Ananias H.J.K., Van Den Heuvel M.C., Helfrich W., De Jong I.J. (2009). Expression of the gastrin-releasing peptide receptor, the prostate stem cell antigen and the prostate-specific membrane antigen in lymph node and bone metastases of prostate cancer. Prostate.

[17] Beer M., Montani M., Gerhardt J., Wild P.J., Hany T.F., Hermanns T., Müntener M., Kristiansen G. (2012). Profiling gastrin-releasing peptide receptor in prostate tissues: Clinical implications and molecular correlates. Prostate.

[18] Baratto L., Duan H., Maecke H.R., Iagaru A. (2020). Imaging the Distribution of Gastrin Releasing Peptide Receptors in Cancer. J. Nucl. Med..

[19] Mansi R., Wang X., Forrer F., Kneifel S., Tamma M.L., Waser B., Cescato R., Reubi J.C., Maecke H.R. (2009). Evaluation of a 1,4,7,10-tetraazacyclododecane-1,4,7,10-tetraacetic acid-conjugated bombesin-based radioantagonist for the labeling with single-photon emission computed tomography, positron emission tomography, and therapeutic radionuclides. Clin. Cancer Res..

[20] Baratto L., Jadvar H., Iagaru A. (2017). Prostate Cancer Theranostics Targeting Gastrin-Releasing Peptide Receptors. Mol. Imaging Biol..

[21] Roivainen A., Kähkönen E., Luoto P., Borkowski S., Hofmann B., Jambor I., Lehtiö K., Rantala T., Rottmann A., Sipilä H. (2013). Plasma pharmacokinetics, whole-body distribution, metabolism, and radiation dosimetry of ^68^Ga bombesin antagonist BAY 86-7548 in healthy men. J. Nucl. Med..

[22] Kahkonen E., Jambor I., Kemppainen J., Lehtio K., Gronroos T.J., Kuisma A., Luoto P., Sipila H.J., Tolvanen T., Alanen K. (2013). In Vivo imaging of prostate cancer using [^68^Ga]-labeled bombesin analog BAY86-7548. Clin. Cancer Res..

[23] Minamimoto R., Hancock S., Schneider B., Chin F.T., Jamali M., Loening A., Vasanawala S., Gambhir S.S., Iagaru A. (2016). Pilot comparison of ^68^Ga-RM2 PET and ^68^Ga-PSMA-11 PET in patients with biochemically recurrent prostate cancer. J. Nucl. Med..

[24] Mannweiler S., Amersdorfer P., Trajanoski S., Terrett J.A., King D., Mehes G. (2009). Heterogeneity of prostate-specific membrane antigen (PSMA) expression in prostate carcinoma with distant metastasis. Pathol. Oncol. Res..

[25] Eder M., Schäfer M., Bauder-Wüst U., Haberkorn U., Eisenhut M., Kopka K. (2014). Preclinical evaluation of a bispecific low-molecular heterodimer targeting both PSMA and GRPR for improved PET imaging and therapy of prostate cancer. Prostate.

[26] Bandari R.P., Jiang Z., Reynolds T.S., Bernskoetter N.E., Szczodroski A.F., Bassuner K.J., Kirkpatrick D.L., Rold T.L., Sieckman G.L., Hoffman T.J. (2014). Synthesis and biological evaluation of copper-64 radiolabeled [DUPA-6-Ahx-(NODAGA)-5-Ava-BBN(7-14)NH2], a novel bivalent targeting vector having affinity for two distinct biomarkers (GRPr/PSMA) of prostate cancer. Nucl. Med. Biol..

[27] Liolios C., Schäfer M., Haberkorn U., Eder M., Kopka K. (2016). Novel Bispecific PSMA/GRPr Targeting Radioligands with Optimized Pharmacokinetics for Improved PET Imaging of Prostate Cancer. Bioconjug. Chem..

[28] Escudero-Castellanos A., Ocampo-García B., Ferro-Flores G., Santos-Cuevas C., Morales-Ávila E., Luna-Gutiérrez M., Isaac-Olivé K. (2018). Synthesis and preclinical evaluation of the ^177^Lu-DOTA-PSMA(inhibitor)-Lys^3^-bombesin heterodimer designed as a radiotheranostic probe for prostate cancer. Nucl. Med. Commun..

[29] Mendoza-Figueroa M.J., Escudero-Castellanos A., Ramirez-Nava G.J., Ocampo-García B.E., Santos-Cuevas C.L., Ferro-Flores G., Pedraza-Lopez M., Avila-Rodriguez M.A. (2018). Preparation and preclinical evaluation of ^68^Ga-iPSMA-BN as a potential heterodimeric radiotracer for PET-imaging of prostate cancer. J. Radioanal. Nucl. Chem..

[30] Abouzayed A., Yim C.-B., Mitran B., Rinne S.S., Tolmachev V., Larhed M., Rosenström U., Orlova A. (2019). Synthesis and Preclinical Evaluation of Radio-Iodinated GRPR/PSMA Bispecific Heterodimers for the Theranostics Application in Prostate Cancer. Pharmaceutics.

[31] Mitran B., Varasteh Z., Abouzayed A., Rinne S.S., Puuvuori E., De Rosa M., Larhed M., Tolmachev V., Orlova A., Rosenström U. (2019). Bispecific GRPR-Antagonistic Anti-PSMA/GRPR Heterodimer for PET and SPECT Diagnostic Imaging of Prostate Cancer. Cancers.

[32] Lundmark F., Abouzayed A., Mitran B., Rinne S.S., Varasteh Z., Larhed M., Tolmachev V., Rosenström U., Orlova A. (2020). Heterodimeric Radiotracer Targeting PSMA and GRPR for Imaging of Prostate Cancer—Optimization of the Affinity towards PSMA by Linker Modification in Murine Model. Pharmaceutics.

[33] Bandari R.P., Carmack T.L., Malhotra A., Watkinson L., Fergason Cantrell E.A., Lewis M.R., Smith C.J. (2021). Development of Heterobivalent Theranostic Probes Having High Affinity/Selectivity for the GRPR/PSMA. J. Med. Chem..

[34] Ye S., Li H., Hu K., Li L., Zhong J., Yan Q., Wang Q. (2022). Radiosynthesis and biological evaluation of 18F-labeled bispecific heterodimer targeted dual gastrin-releasing peptide receptor and prostate-specific membrane antigen for prostate cancer imaging. Nucl. Med. Commun..

[35] Rivera-Bravo B., Ramírez-Nava G., Mendoza-Figueroa M.J., Ocampo-García B., Ferro-Flores G., Ávila-Rodríguez M.A., Santos-Cuevas C. (2021). [^68^Ga]Ga-iPSMA-Lys^3^-Bombesin: Biokinetics, dosimetry and first patient PET/CT imaging. Nucl. Med. Biol..

[36] Martiniova L., De Palatis L., Etchebehere E., Ravizzini G. (2016). Gallium-68 in Medical Imaging. Curr. Radiopharm..

[37] Capala J., Turkbey B., Tagawa S.T., Bouchelouche K., Choyke P., Goldsmith S.J. (2010). PET/CT Imaging and Radioimmunotherapy of Prostate Cancer. Semin. Nucl. Med..

[38] Solomon B., McArthur G.A., Cullinane C., Zalcberg J.R., Hicks R.J. (2003). Applications of Positron Emission Tomography in the Development of Molecular Targeted Cancer Therapeutics. BioDrugs.

[39] (1996). The Guide for Care and Use of Laboratory Animals.

[40] Shekunova E.V., Kovaleva M.A., Makarova M.N., Makarov V.G. (2020). Dose selection in preclinical studies: Cross-species dose conversion. Bull. Sci. Cent. Expert Eval. Med. Prod..

[41] Saad F. (2019). Quality of life in men with prostate cancer. Lancet Oncol..

[42] Debnath S., Zhou N., McLaughlin M., Rice S., Pillai A.K., Hao G., Sun X. (2022). PSMA-Targeting Imaging and Theranostic Agents—Current Status and Future Perspective. Int. J. Mol. Sci..

[43] Mapelli P., Ghezzo S., Samanes Gajate A.M., Preza E., Brembilla G., Cucchiara V., Ahmed N., Bezzi C., Presotto L., Bettinardi V. (2021). Preliminary Results of an Ongoing Prospective Clinical Trial on the Use of 68 Ga-PSMA and 68 Ga-DOTA-RM2 PET/MRI in Staging of High-Risk Prostate Cancer Patients. Diagnostics.

[44] Hörmann A.A., Klingler M., Rangger C., Mair C., Decristoforo C., Uprimny C., Virgolini I.J., von Guggenberg E. (2021). Radiopharmaceutical Formulation and Preclinical Testing of ^68^Ga-Labeled DOTA-MGS5 for the Regulatory Approval of a First Exploratory Clinical Trial. Pharmaceuticals.

[45] Zhu W., Cheng Y., Jia R., Zhao H., Bai C., Xu J., Yao S., Huo L. (2021). A Prospective, Randomized, Double-Blind Study to Evaluate the Safety, Biodistribution, and Dosimetry of ^68^Ga-NODAGA-LM_3_ and ^68^Ga-DOTA-LM_3_ in Patients with Well-Differentiated Neuroendocrine Tumors. J. Nucl. Med..

[46] Ballal S., Yadav M.P., Moon E.S., Kramer V.S., Roesch F., Kumari S., Tripathi M., ArunRaj S.T., Sarswat S., Bal C. (2021). Biodistribution, pharmacokinetics, dosimetry of [^68^Ga]Ga-DOTA.SA.FAPi, and the head-to-head comparison with [^18^F]F-FDG PET/CT in patients with various cancers. Eur. J. Nucl. Med. Mol. Imaging.

[47] Giesel F.L., Adeberg S., Syed M., Lindner T., Jiménez-Franco L.D., Mavriopoulou E., Staudinger F., Tonndorf-Martini E., Regnery S., Rieken S. (2021). FAPI-74 PET/CT Using Either ^18^F-AlF or Cold-Kit ^68^Ga Labeling: Biodistribution, Radiation Dosimetry, and Tumor Delineation in Lung Cancer Patients. J. Nucl. Med..

[48] Bakker I.L., Fröberg A.C., Busstra M.B., Verzijlbergen J.F., Konijnenberg M., van Leenders G.J.L.H., Schoots I.G., de Blois E., van Weerden W.M., Dalm S.U. (2021). GRPr antagonist ^68^Ga-SB_3_ PET/CT-imaging of primary prostate cancer in therapy-naive patients. J. Nucl. Med..

[49] Zhang J., Niu G., Fan X., Lang L., Hou G., Chen L., Wu H., Zhu Z., Li F., Chen X. (2018). PET using a GRPR antagonist ^68^Ga-RM26 in healthy volunteers and prostate cancer patients. J. Nucl. Med..

