# Supplementary material for: Preclinical Characterisation of PSMA/GRPR-Targeting Heterodimer [68Ga]Ga-BQ7812 for PET Diagnostic Imaging of Prostate Cancer: A Step towards Clinical Translation
Source: Cancers (Basel). 2023 Jan 10;15(2):442. doi: 10.3390/cancers15020442 (PMC9856709; doi:10.3390/cancers15020442)
Supplement: Supplementary file 1 [file cancers-15-00442-s001.zip › cancers-2122113-supplementary.pdf]

Supplementary material

Preclinical Characterisation of PSMA/GRPR-targeting Heterodimer [^{68}Ga]Ga-BQ7812 for PET Diagnostic Imaging of Prostate Cancer: a Step towards Clinical Translation

Fanny Lundmark [1], Ayman Abouzayed [1], Sara S. Rinne [1], Vasiliy Timofeev [2], Nadezhda Sipkina [2], Maria Naan [2], Anastasia Kirichenko [3], Maria Vasyutina [3], Daria Ryzhkova [4], Ulrika Rosenström [1] and Anna Orlova [1,5,*]

[1] Department of Medicinal Chemistry, Uppsala University, 751 23 Uppsala, Sweden
[2] Personalized Medicine Centre, Almazov National Medical Research Centre, 2 Akkuratova Str., 197341 Saint Petersburg, Russia
[3] Preclinical and Translational Research Centre, Almazov National Medical Research Centre, 2 Akkuratova Str., 197341 Saint Petersburg, Russia
[4] Department of Nuclear Medicine and Radiation Technology with Clinic, Almazov National Medical Research Centre, 2 Akkuratova Str., 197341 Saint Petersburg, Russia
[5] Science for Life Laboratory, Department of Medicinal Chemistry, Uppsala University, 751 23 Uppsala, Sweden
* Correspondence: anna.orlova@ilk.uu.se

General information

Instruments and Equipment

Analytical high performance liquid chromatography (HPLC) was performed on a Dionex UltiMate 3000 HPLC system with a Bruker amazon SL ion trap mass spectrometer and detection by UV (diode array detector, 214, 254, and 280 nm) and electrospray ionization (ESI) MS using a Penomenex Kinetex C18 column (50 × 3.0 mm, 2.6 μm particle size, 100 Å pore size) with gradients of $H_2O/CH_3CN/0.05\%HCOOH$ as mobile phase at a flow rate of 1.5 mL/min. Preparative reversed phase high-performance liquid chromatography (RP-HPLC) was performed by UV-trigged (254 nm) fraction collection with a Glison HPLC system using a Machery-nagel NUCLEODUR C18 HTec column (21 × 125 mm, particle size 5 μm) and $H_2O/CH_3CN/0.1\%TFA$ as mobile phase at a flow rate of 10 mL/min. $^{68}Ge/Ga$-generator (Cyclotron Co., Obninsk, Russia) using 0.1 M metal-free hydrochloric acid was used to elute gallium-68. Radioactivity content was measured using an automated gamma counter (3-inch NaI(Tl) detector, 2480 Wizard2, PerkinElmer). nanoPETimaging of PC3-pip xenografted mice using a nanoScan PET/MR1 3T camera (Mediso Medical Imaging System Ltd., Budapest, Hungary) and CT scan was performed using a nanoScan SPECT/CT camera (Mediso Medical Imaging System Ltd., Budapest, Hungary). Reconstruction of the PET/CT scans was done using Nucline nanoScan 3.04.014.0000 software. The absorbed doses in the dosimetry investigation were estimated using OLINDA/EXM 1.0. Statistical analysis was performed by one-way ANOVA with Bonferroni's test corrected for multiple comparisons using GraphPad Prism 8 for Windows (GraphPad Software Inc, San Diego, CA, USA), p-values <0.05 were considered statistical significant.

Chemicals and Solvents

Starting materials and solvents were purchased from Sigma Aldrich, Fisher Scientific, and Honeywell, and used without further purification if nothing else stated. NOTA-bis(tBu)-ester was purchased from CheMatech, France; Fmoc Rink Amide MBHA resin (loading 0.69 mmol/g) and L-Glu(OBn)-O(tBu) were purchased from Iris Biotech Gmbh (Marktredwitz, Germany).

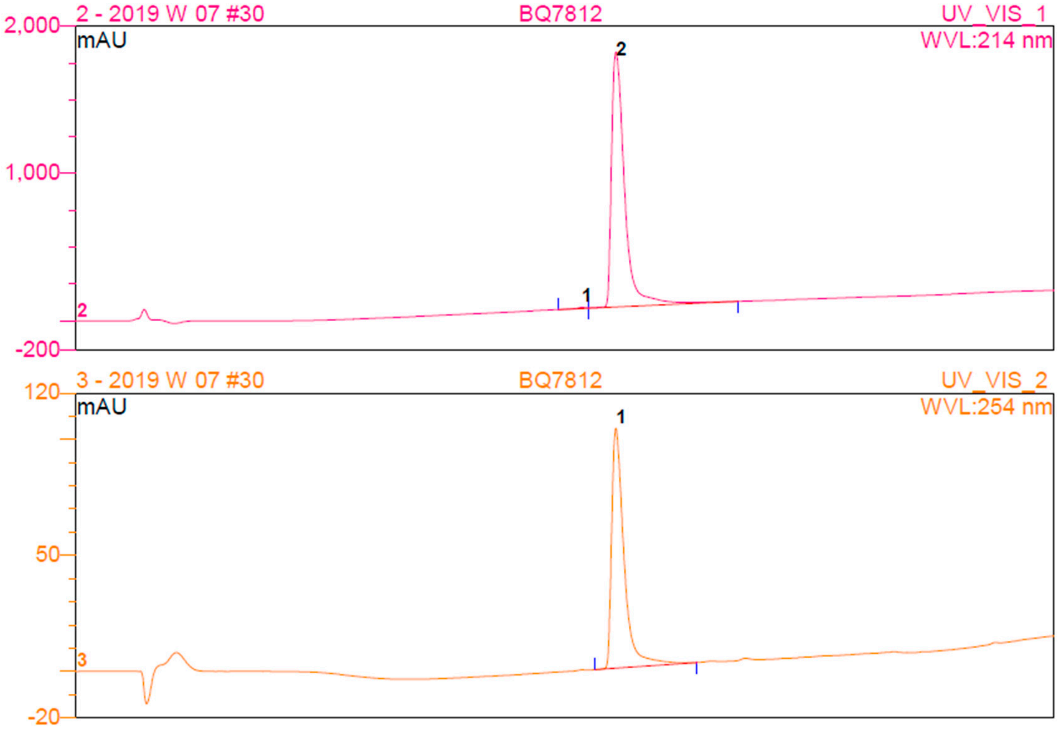

Figure S1. Analytical HPLC chromatogram of BQ7812. UV-detection at 214 and 254 nm.

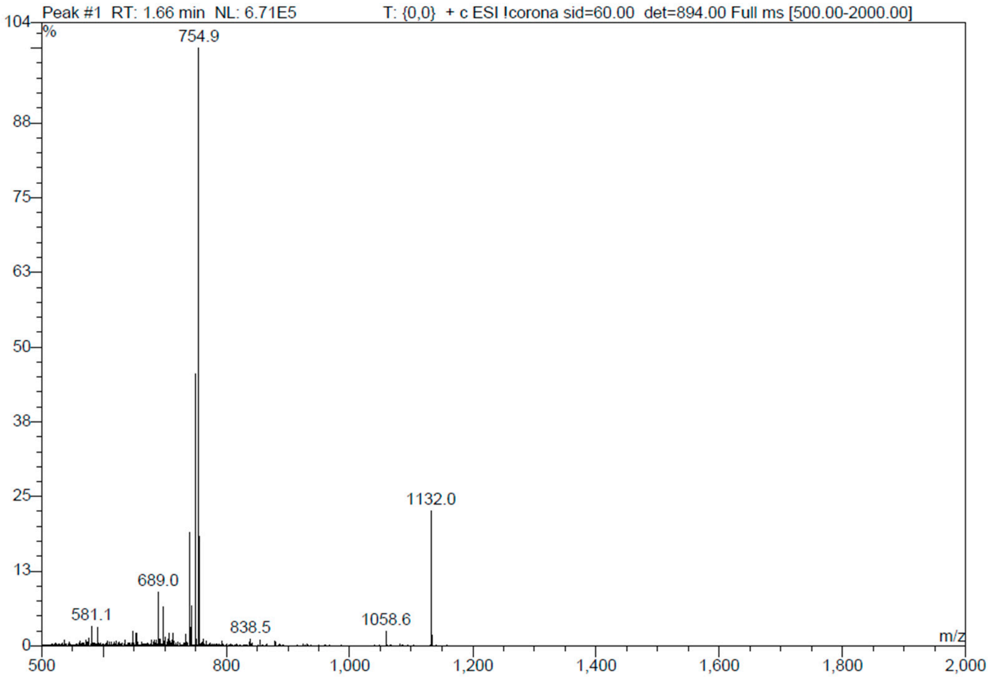

Figure S2. MS-chromatogram of BQ7812 with observed $[M+2H]^{2+}$ = 1132.0 and $[M+3H]^{3+}$ = 754.9.

Table S1. Ex vivo biodistribution of [^{68}Ga]Ga-BQ7812 (40 pmol/animal, 30 kBq) in BALB/c nu/nu PC3-pip xenografted tumour bearing mice at 1 and 3 h pi. Data are presented as average ± standard deviation. To the left; Uptake of activity was calculated as percent injected dose per tissue weight (% ID/g), To the right; tumour-to-organ ratios. Statistical analysis was done using one-way ANOVA with Bonferroni's test corrected for multiple comparisons.

	Uptake of [^{68}Ga]Ga-BQ7812 (%IA/g)				Tumour-to-organ ratio (T/O)	
	1 h pi			3 h pi	1 h pi	3 h pi
	Non-blocked	GRPR blocked	PSMA blocked	Non-blocked	Non-blocked	Non-blocked
**Blood**	0.86 ± 0.16	0.69 ± 0.28	0.80 ± 0.24	0.16 ± 0.02^{c}	13 ± 3	35 ± 4^{c}
**Salivary Glands**	0.44 ± 0.07	0.29 ± 0.07^{a}	0.38 ± 0.12	0.14 ± 0.03^{c}	24 ± 3	40 ± 8^{c}
**Lungs**	0.86 ± 0.15	-----	-----	0.20 ± 0.05^{c}	12 ± 3	29 ± 5^{c}
**Liver**	0.89 ± 0.10	-----	-----	0.57 ± 0.03^{c}	11.8 ± 1.0	9.9 ± 0.8
**Spleen**	0.92 ± 0.16	-----	-----	0.33 ± 0.03^{c}	12 ± 3	18 ± 3
**Pancreas**	5.63 ± 0.58	0.76 ± 0.14^{a}	6.33 ± 1.86	0.60 ± 0.16^{c}	1.9 ± 0.2	10 ± 4^{c}
**Small Intestine**	1.33 ± 0.31	-----	-----	0.20 ± 0.02^{c}	8 ± 2	28 ± 4^{c}
**Kidneys**	45.26 ± 16.55	34.50 ± 8.74	13.78 ± 0.45^{b}	10.55 ± 2.13^{c}	0.3 ± 0.1	0.53 ± 0.07^{c}
**Tumour (PC3-pip)**	10.44 ± 0.97	5.73 ± 0.72^{a}	8.26 ± 1.03^{b}	5.68 ± 0.62^{c}	-----	-----
**Muscle**	0.21 ± 0.04	-----	-----	0.05 ± 0.01^{c}	52 ± 12	116 ± 22^{c}
**Bone**	0.31 ± 0.06	-----	-----	0.13 ± 0.03^{c}	35 ± 8	47 ± 13

a Value in GRPR-blocked group is significantly different than in Non-blocked group;
b Value in PSMA-blocked group is significantly different than in Non-blocked group;
c Value in Non-blocked group 3 h pi is significantly different than in Non-blocked group 1 h pi.
